# Comparison of Glidescope^®^ Go^™^, King Vision^™^, Dahlhausen VL, I‑View^™^ and Macintosh laryngoscope use during difficult airway management simulation by experienced and inexperienced emergency medical staff: A randomized crossover manikin study

**DOI:** 10.1371/journal.pone.0236474

**Published:** 2020-07-30

**Authors:** Andreas Moritz, Veronika Leonhardt, Johannes Prottengeier, Torsten Birkholz, Joachim Schmidt, Andrea Irouschek

**Affiliations:** Department of Anesthesiology, University Hospital Erlangen, Faculty of Medicine, Friedrich-Alexander-Universität (FAU) Erlangen-Nürnberg, Erlangen, Germany; University of Alberta, CANADA

## Abstract

**Background:**

In pre-hospital emergency care, video laryngoscopes (VLs) with disposable blades are preferably used due to hygienic reasons. However, there is limited existing data on the use of VLs with disposable blades by emergency medical staff. Therefore, the aim of this study was to compare the efficacy of four different VLs with disposable blades and the conventional standard Macintosh laryngoscope, when used by anesthetists with extensive previous experience and paramedics with little previous experience in endotracheal intubation (ETI) in a simulated difficult airway.

**Methods:**

Fifty-eight anesthetists and fifty-four paramedics participated in our randomized crossover manikin trial. Each performed ETI with the new Glidescope^®^ Go^™^, the Dahlhausen VL, the King Vision^™^, the I-View^™^ and the Macintosh laryngoscope. “Time to intubate” was the primary endpoint. Secondary endpoints were “time to vocal cords”, “time to ventilate”, overall success rate, number of intubation attempts and optimization maneuvers, Cormack-Lehane score, severity of dental compression and subjective impressions.

**Results:**

The Glidescope^®^ Go^™^, the Dahlhausen VL and the King Vision^™^ provided superior intubation conditions in both groups without affecting the number of intubation attempts or the time required for successful intubation. When used by anesthetists with extensive experience in ETI, the use of VLs did not affect the overall success rate. In the hands of paramedics with little previous experience in ETI, the failure rate with the Macintosh laryngoscope (14.8%) decreased to 3.7% using the Glidescope^®^ Go^™^ and the Dahlhausen VL. Despite the advantages of hyperangulated video laryngoscopes, the I-View^™^ performed worst.

**Conclusions:**

VLs with hyperangulated blades facilitated ETI in both groups and decreased the failure rate by an absolute 11.1% when used by paramedics with little previous experience in ETI. Our results therefore suggest that hyperangulated VLs could be beneficial and might be the method of choice in comparable settings, especially for emergency medical staff with less experience in ETI.

## Introduction

Airway management is one of the major challenges of pre-hospital emergency medicine. However, various parameters relating to the patient (anatomical or physiological factors), environment (confined space, poor lighting conditions), out-of-hospital team work and the need for immediate decision-making may contribute to potential difficulties [1, 2]. Thus, in pre-hospital emergency settings, advanced airway management of critically ill or injured patients still remains a challenge, even in the hands of experienced emergency physicians. A difficult airway has been reported in up to 14.8% of patients managed by anesthesia trained physicians in a pre-hospital setting [3]. Several studies demonstrated an incidence of failed pre-hospital endotracheal intubation (ETI) in physician-staffed emergency medical systems varying from 1 to 2% [3–5]. In non-physician-manned emergency medical services, ETI failure rates of even 15–23% have been reported [4, 6]. Difficult or failed tracheal intubation is well-recognized as an important cause of morbidity and mortality associated with anesthesia and emergency medicine [7]. In addition, multiple intubation attempts have been shown to correlate with an increased incidence of adverse events [8], delay in transport, prolonged hospitalization, worse neurologic outcomes [9] and increased mortality [10].

In recent years, the implementation of video laryngoscopy has improved the in-hospital management of the difficult airway. In a Cochrane systematic review of video laryngoscope (VL) use in patients during general anesthesia, Lewis and colleagues reported reduced numbers of failed intubations, improved laryngeal view, facilitated handling and reduced airway trauma. However, not all VLs perform equally and, in addition, the intubation success also depends on the experience of the user with the respective device [11].

In pre-hospital emergency care, laryngoscopes with disposable blades are preferably used due to hygienic reasons [12–15]. Although the equipment for emergency intubation of critically ill patients should meet the highest requirements [2], no study has yet evaluated the performance of video laryngoscopes with disposable blades when used by experienced and inexperienced emergency medical staff. Thus, the aim of our prospective randomized crossover study was to compare the efficacy of four different VLs with disposable blades (the new Glidescope^®^ Go^™^, the Dahlhausen VL, the King Vision^™^ aBlade^™^, the I-View^™^) and the conventional standard Macintosh laryngoscope, when used by anesthetists with extensive previous experience in conventional and video laryngoscopy and paramedics with little previous experience in conventional laryngoscopy and even less previous experience in the use of video laryngoscopy in a simulated difficult airway scenario.

## Materials and methods

### Study design and setting

The manikin study (randomized crossover design) was evaluated and approved by the institutional ethics committee (Ethics Committee of the Friedrich-Alexander-Universität Erlangen-Nürnberg; reference number: 408_18 B). Following written informed consent, fifty-eight anesthetists and fifty-four paramedics were recruited to the study. Data were anonymized and information on the performance of individual participants was not made available to anybody outside the research team.

**Fig 1:**
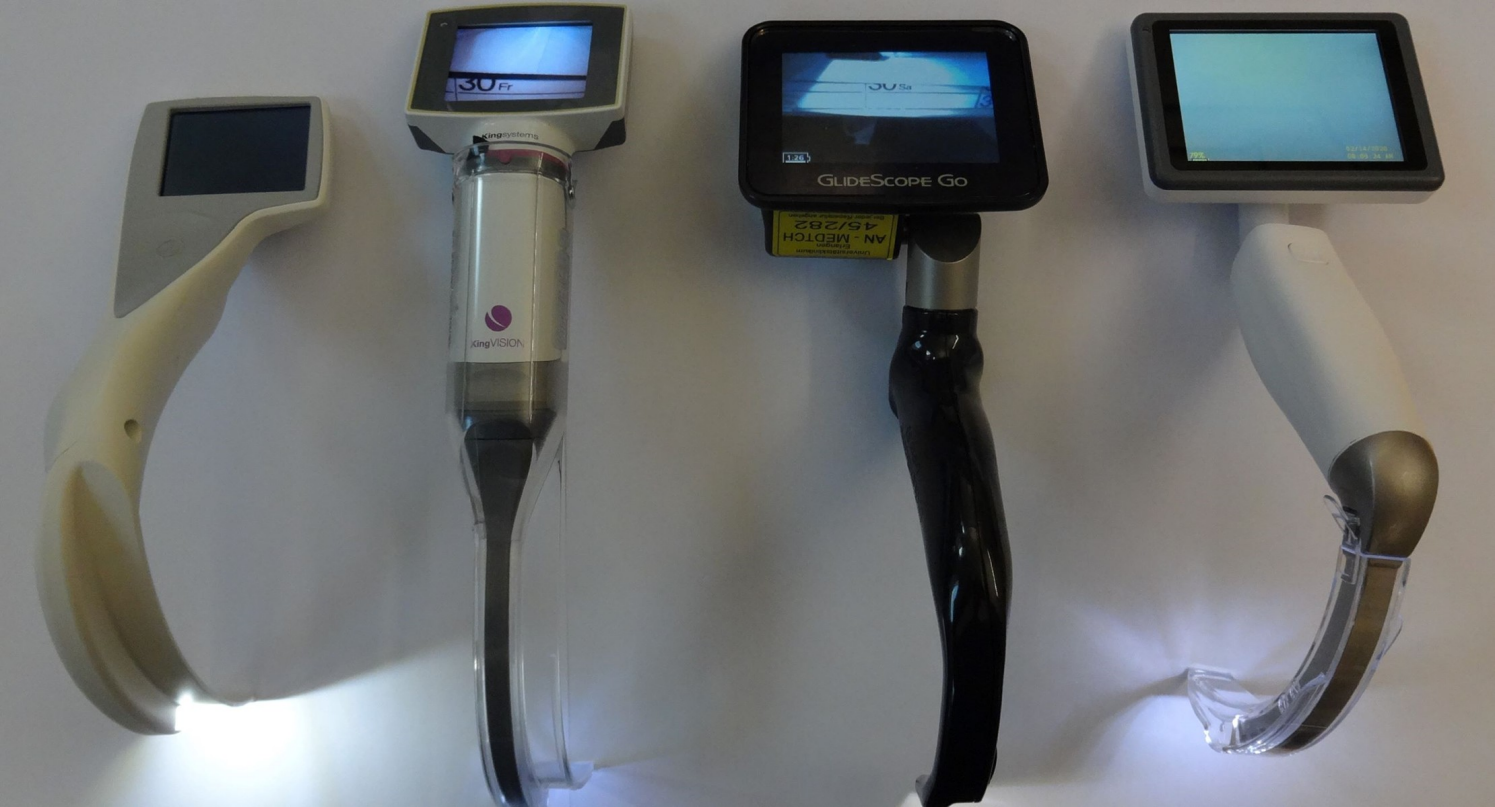
The four different video laryngoscopes (VLs) used in this study. From left to right: I-View^™^ one-size single-use VL (Intersurgical Ltd., Wokingham, Berkshire, UK); King Vision^™^ aBlade^™^ VL with a non-channeled aBlade single-use blade size 3 (Ambu A/S, Ballerup, Denmark); Glidescope^®^ Go^™^ VL with a LoPro S3 single-use blade (Verathon Inc., Bothell, WA, USA); Dahlhausen VL for single-use blades (Dahlhausen VL) with a MAC DX3 single-use blade (P. J. Dahlhasuen & Co. GmbH, Köln, Germany).

**Fig 2:**
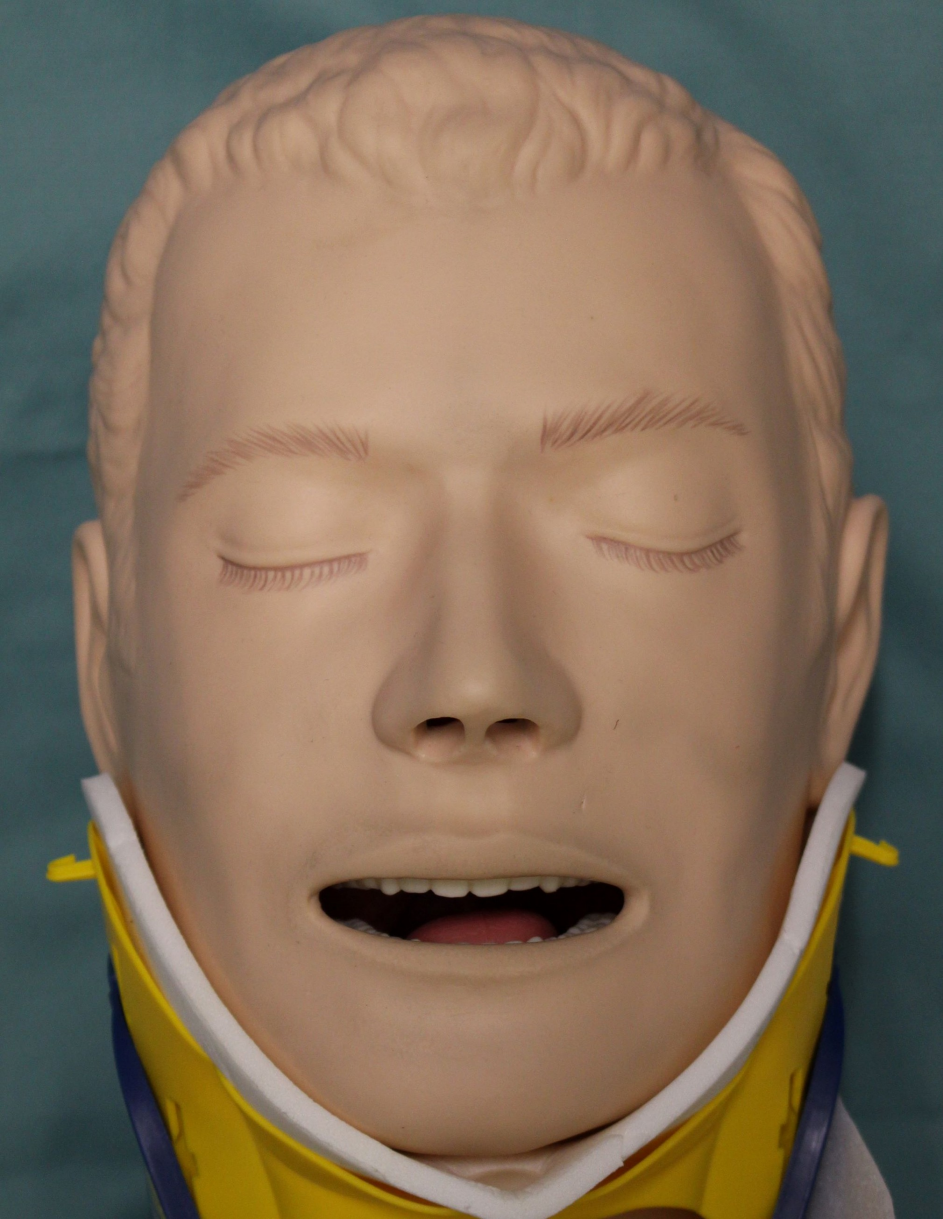
Laerdal airway management trainer. The difficult airway is simulated by cervical immobilization applying a cervical collar.

Each participant performed ETI with the Glidescope^®^ Go^™^ VL, a new portable high-resolution video laryngoscopy system, with a LoPro S3 single-use blade (Verathon Inc., Bothell, WA, USA), the Dahlhausen VL for single-use blades with a MAC DX3 single-use blade (P. J. Dahlhasuen & Co. GmbH, Köln, Germany), the King Vision^™^ aBlade^™^ VL with a non-channeled aBlade single-use blade size 3 (Ambu A/S, Ballerup, Denmark), the I-View^™^ one-size single-use VL (Intersurgical Ltd., Wokingham, Berkshire, UK) (Fig 1) and a conventional standard single-use Macintosh laryngoscope blade size 3 (PROACT HPC^™^ Green System Laryngoscope Blade-MAC 3, PROACT Medical Ltd., Corby, Northamptonshire, UK; Heine Standard F.O. 4 LED NT metallic laryngoscope handle, Heine Optotechnik GmbH & Co. KG, Herrsching, Germany) in a difficult airway manikin (Laerdal Airway Management Trainer, Laerdal Medical AS, Stavanger, Norway) (Fig 2). The neck of the manikin was fixed in a neutral position by a rigid cervical collar. Thus, the distance between the free edge of the upper and lower incisors (interdental distance) was limited, fulfilling conditions for a difficult intubation model [16, 17].

Randomized order of application was provided by five sealed opaque envelopes each containing a different device name.

All intubations were performed with a 7.0 mm cuffed endotracheal tube (ETT; Rüsch Super Safety Clear endotracheal tube, I.D. 7.0 mm, Teleflex Medical GmbH, Fellbach, Germany). The cuff was lubricated with a silicone spray and was inflated and deflated with a 10 ml syringe. A reusable intubation stylet (Teleflex medical GmbH, Fellbach, Germany) was used to facilitate intubation with the Macintosh laryngoscope. For ETI with the Glidescope^®^ Go^™^, the Dahlhausen VL, the King Vision^™^ and the I-View^™^ a rigid intubation stylet (GlideRite Rigid Stylet, Verathon Inc., Bothell, WA, USA) was used.

### Measurements

#### Objective findings

The primary endpoint was the “time to intubate”. Esophageal intubations, attempts requiring more than 120 seconds or more than two attempts (withdrawal of the device from the mouth followed by repositioning) were recorded as failure to intubate. Stopwatch studies were made by a single person having direct observation to avoid interobserver error.

In order to compare the different intubation devices, the intubation process was divided into different time episodes:

1. The time to visualization of the glottis (“time to vocal cords”) was defined as the time from insertion of the blade between the teeth until the glottis was visualized.
2. The time to tracheal intubation (“time to intubate”) was defined as the time from insertion of the blade between the teeth until the ETT was deemed to be positioned correctly by each participant.
3. The duration of a successful intubation attempt was defined as the time from insertion of the blade between the teeth until the ETT was connected to a self-inflating resuscitation bag and lung inflation was confirmed (“time to ventilate”).

We recorded the rate of successful intubation, the number of intubation attempts and the laryngeal view according to the Cormack-Lehane score [18]. The number of optimization maneuvers (readjustment of the head position, application of external laryngeal pressure and the need for assistance by a second person) and the number of audible dental click sounds indicating dental damage were recorded as 0, 1, and ≥ 2 times during ETI.

#### Subjective findings

After completing the procedure, each anesthetist was asked to score

- the view
- the handling
- the stability
- the force applied during tracheal intubation
- the difficulty of tracheal intubation

using a numeric rating scale (NRS) (0 to 100 mm, from excellent/very easy to poor/very difficult). Finally, the participants were asked to indicate their preferred intubation device.

### Data analysis

The sample size was calculated using G*Power (Version 3.1.9.4, Faul F., 2019, Germany). Based on “time to intubate”-duration compiled in a pilot study, an effect size f of 0.39 was anticipated. Considering an α error of 0.05 and β error of 0.1, sample size calculation results that at least 50 experienced and 50 inexperienced participants would be required.

All the statistical analyses were performed using SPSS software (IBM^®^ SPSS^®^ Statistics, version 26.0, IBM Corp., Armonk, NY, USA). Data for the rate of successful intubation were analyzed using the Cochran`s Q test followed by Dunn-Bonferroni post-hoc tests. Data for the “time to vocal cords”, the “time to intubate”, the “time to ventilate”, the number of intubation attempts, the number of optimization maneuvers, the number of audible dental clicks, the Cormack-Lehane score, the view, the handling, the stability, the force applied during tracheal intubation and the difficulty of tracheal intubation were analyzed using paired non-parametric tests. The Friedman and Wilcoxon signed-rank tests with Bonferroni correction were used for multiple and post-hoc comparisons respectively. Statistical significance was accepted at p < 0.05.

## Results

### Participant characteristics

Fifty-eight anesthetists (43 [74%] senior house officers, 11 [19%] specialist registrars, 4 [7%] consultants) and fifty-four paramedics participated in this randomized crossover trial. The anesthetists included in this study also work as emergency physicians as part of their work in the Department of Anesthesiology at the University Hospital Erlangen. The participant characteristics and the self-reported experience in video and direct laryngoscopy are summarized in Table 1. Data in the result section are presented as mean difference with 95% confidence interval, MD [95% CI].

**Table 1:** Participant characteristics and self-reported estimates of previous experience in conventional and video laryngoscopy.

	Anesthetists	Paramedics
Gender, n/N (%)		
Female	18/58 (31)	15/54 (28)
Male	40/58 (69)	39/54 (72)
Age (y), median (IQR)	32 (29–35.3)	39.5 (28.5–46)
Number of conventional intubations (n), median (IQR)	1000 (600–2000)	10 (3.8–20)
Experience in video laryngoscopy (n), median (IQR)	30 (12.5–80)	0 (0–1)

Data are presented as median (inter-quartile range, IQR) or as fraction, n/N (%).

### Anesthetists with extensive previous experience in ETI

Regarding the primary endpoint (“time to intubate”), the “time to ventilate” and the overall success rate, no significant differences were observed between the conventional Macintosh laryngoscope, the Glidescope^®^ Go^™^, the Dahlhausen VL and the King Vision^™^. However, the Glidescope^®^ Go^™^, the Dahlhausen VL and the King Vision^™^ enabled a significantly shorter “time to vocal cords” (4.1 s [2.0-6.2 s], p < 0.001, Glidescope^®^ Go^™^ vs. Macintosh laryngoscope; 14 s [10.3-17.7 s], p < 0.001, Glidescope^®^ Go^™^ vs. I-View^™^; 5.2 s [2.7-7.7 s], p < 0.001, Dahlhausen VL vs. Macintosh laryngoscope; 14.7 s [11.4-18.1 s], p < 0.001, Dahlhausen VL vs. I-View^™^; 5.4 s [3-7.8 s], p < 0.001, King Vision^™^ vs. Macintosh laryngoscope; 14.5 s [11.2-17.7 s], p < 0.001, King Vision^™^ vs. I-View^™^) and significantly improved the Cormack and Lehane grade (1 [0.8-1.2], p < 0.001, Glidescope^®^ Go^™^ vs. Macintosh laryngoscope; 0.9 [0.7-1.2], p < 0.001, Glidescope^®^ Go^™^ vs. I-View^™^; 0.8 [0.6-1], p < 0.001, Dahlhausen VL vs. Macintosh laryngoscope; 0.7 [0.5-1], p < 0.001, Dahlhausen VL vs. I-View^™^; 0.9 [0.7-1.1], p < 0.001, King Vision^™^ vs. Macintosh laryngoscope; 0.8 [0.6-1.1], p < 0.001, King Vision^™^ vs. I-View^™^) compared to the Macintosh laryngoscope and the I-View^™^, when used by anesthetists with extensive previous experience in direct and in video laryngoscopy. In addition, the severity of dental compression was lower with the Glidescope^®^ Go^™^, the Dahlhausen VL and the King Vision^™^ (0.5 [0.3–0.7], p < 0.001, King Vision^™^ vs. Macintosh laryngoscope; 0.7 [0.4-0.9], p < 0.001, King Vision^™^ vs. I-View^™^; 0.4 [0.2-0.6], p < 0.01, Glidescope^®^ Go^™^ vs. Macintosh laryngoscope; 0.6 [0.4-0.8], p < 0.001, Glidescope^®^ Go^™^ vs. I-View^™^; 0.4 [0.1-0.6], p < 0.05, Dahlhausen VL vs. Macintosh laryngoscope; 0.6 [0.3-0.8], p < 0.01, Dahlhausen VL vs. I-View^™^). Compared to the other devices, the I-View^™^ VL performed significantly worse. The recorded intubation times were significantly increased (“time to vocal cords”: 14 s [10.3-17.7 s], p < 0.001, Glidescope^®^ Go^™^ vs. I-View^™^; 14.5 s [11.2-17.7 s], p < 0.001, King Vision^™^ vs. I-View^™^; 14.7 s [11.4-18.1 s], p < 0.001, Dahlhausen VL vs. I-View^™^; 10.5 s [6.6-14.3 s], p < 0.001, Macintosh laryngoscope vs. I-View^™^; “time to intubate”: 13.2 s [8.2-18.1 s], p < 0.001, Glidescope^®^ Go^™^ vs. I-View^™^; 13 s [8-17.9 s], p < 0.001, King Vision^™^ vs. I-View^™^; 10.2 s [4.3-16.1 s], p < 0.01, Dahlhausen VL vs. I-View^™^; 11 s [5.8-16.2 s], p < 0.01, Macintosh laryngoscope vs. I-View^™^; “time to ventilate”: 12 s [6.9-17 s], p < 0.001, Glidescope^®^ Go^™^ vs. I-View^™^; 13.4 s [8.4-18.4 s], p < 0.001, King Vision^™^ vs. I-View^™^; 11.8 s [6.7-16.9 s], p < 0.01, Macintosh laryngoscope vs. I-View^™^; 9.6 s [3.1-16.2 s], p < 0.05, Dahlhausen VL vs. I-View^™^) and tracheal intubation was less successful (n/N (%): 46/58 (79.3), p < 0.001) compared to the other intubation devices. Although the experienced participants needed more attempts for tracheal intubation using the I-View^™^, this reached statistical significance only when compared to the Dahlhausen VL (0.3 [0.1–0.5], p < 0.05). Post-hoc comparison revealed no significant differences for the number of optimization maneuvers.

Regarding the subjective values, the Glidescope^®^ Go^™^, the Dahlhausen VL and the King Vision^™^ enabled a significant better view (3.4 cm [2.6-4.1 cm], p < 0.001, Glidescope^®^ Go^™^ vs. Macintosh laryngoscope; 5.3 cm [4.4-6.2 cm], p < 0.001, Glidescope^®^ Go^™^ vs. I-View^™^; 2.8 cm [2-3.6 cm], p < 0.001, Dahlhausen VL vs. Macintosh laryngoscope; 4.7 cm [3.8-5.6 cm], p < 0.001, Dahlhausen VL vs. I-View^™^; 2.4 cm [1.4-3.4 cm], p < 0.001, King Vision^™^ vs. Macintosh laryngoscope; 4.3 cm [3.5-5.1 cm], p < 0.001, King Vision^™^ vs. I-View^™^) were considered to be more stable (1.7 cm [1-2.4 cm], p < 0.001, Glidescope^®^ Go^™^ vs. Macintosh laryngoscope; 1.9 cm [1.1-2.6 cm], p < 0.001, Glidescope^®^ Go^™^ vs. I-View^™^; 1.4 cm [0.7-2.1 cm], p < 0.01, Dahlhausen VL vs. Macintosh laryngoscope; 1.6 cm [0.9-2.3 cm], p < 0.01, Dahlhausen VL vs. I-View^™^; 1.1 cm [0.4-1.8 cm], p < 0.05, King Vision^™^ vs. Macintosh laryngoscope; 1.3 cm [0.6-2 cm], p < 0.05, King Vision^™^ vs. I-View^™^) and required less force during tracheal intubation (3.3 cm [2.7-3.9 cm], p < 0.001, Glidescope^®^ Go^™^ vs. Macintosh laryngoscope; 4.2 cm [3.6-4.8 cm], p < 0.001, Glidescope^®^ Go^™^ vs. I-View^™^; 3.1 cm [2.5-3.8 cm], p < 0.001, Dahlhausen VL vs. Macintosh laryngoscope; 4 cm [3.4-4.6 cm], p < 0.001, Dahlhausen VL vs. I-View^™^; 3.2 cm [2.6-3.9 cm], p < 0.001, King Vision^™^ vs. Macintosh laryngoscope; 4.1 cm [3.4-4.8 cm], p < 0.001, King Vision^™^ vs. I-View^™^) compared to the Macintosh laryngoscope and the I-View^™^. Furthermore, the ETI was judged to be easier using the Glidescope^®^ Go^™^, the Dahlhausen VL and the King Vision^™^ (2.3 cm [1.6-3.1 cm], p < 0.001, Glidescope^®^ Go^™^ vs. Macintosh laryngoscope; 4 cm [3.4-4.7 cm], p < 0.001, Glidescope^®^ Go^™^ vs. I-View^™^; 2.3 cm [1.6-3 cm], p < 0.001, Dahlhausen VL vs. Macintosh laryngoscope; 4 cm [3.3-4.8 cm], p < 0.001, Dahlhausen VL vs. I-View^™^; 2.4 cm [1.7-3.1 cm], p < 0.001, King Vision^™^ vs. Macintosh laryngoscope; 4.1 cm [3.4-4.7 cm], p < 0.001, King Vision^™^ vs. I-View^™^). The I-View^™^ VL was most difficult to handle compared to the other intubation devices (4.4 cm [3.6-5.1 cm], p < 0.001, Glidescope^®^ Go^™^ vs. I-View^™^; 3.6 cm [2.8-4.4 cm], p < 0.001, King Vision^™^ vs. I-View^™^; 3.7 cm [2.9-4.5 cm], p < 0.001, Dahlhausen VL vs. I-View^™^; 4.4 cm [3.6-5.3 cm], p < 0.001, Macintosh laryngoscope vs. I-View^™^). Regarding view (2 cm [1-2.9 cm], p < 0.01) and difficulty of tracheal intubation (1.7 cm [0.9-2.5 cm], p < 0.001), the I-View^™^ was rated even worse than the Macintosh laryngoscope. The Glidescope^®^ Go^™^ was rated best and offered an even better view than the King Vision (1 cm [0.3-1.6 cm], p < 0.05).

Forty percent of the anesthetists with extensive previous experience in ETI preferred the Glidescope^®^ Go^™^, followed by the Dahlhausen VL (31%) and the King Vision^™^ (17%). Only 9% preferred the standard Macintosh laryngoscope and none of the experienced participants would use the I-View^™^.

**Fig 3:**
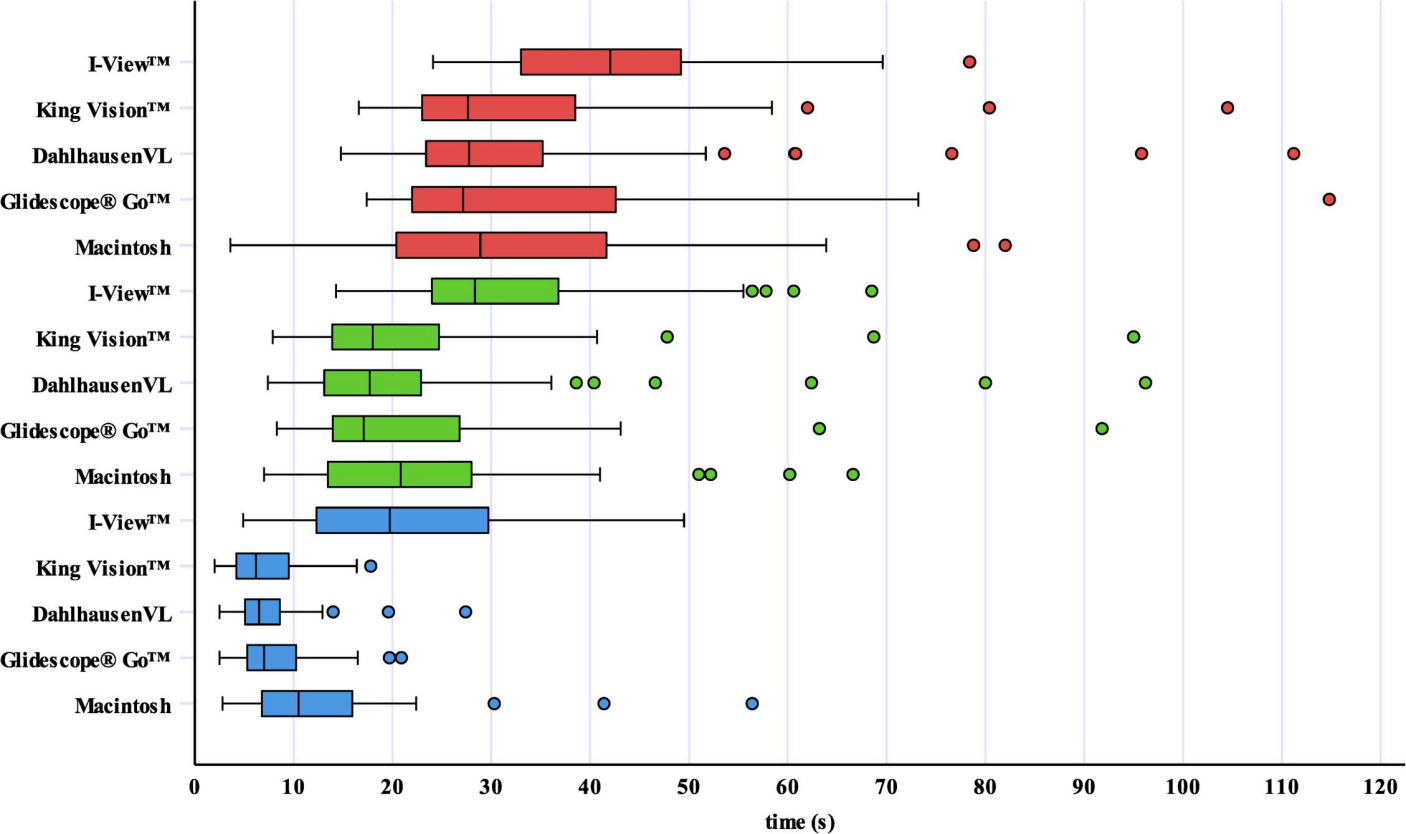
Recorded intubation times of the anesthetists with extensive previous experience in conventional and video laryngoscopy. Data are presented as boxplots that indicate the median (middle line), the 25^th^ and the 75^th^ percentile (box boundaries), the upper and lower limits within 1.5 times inter-quartile range (whiskers) and outliers (circles). The blue boxplots represent the “time to vocal cords”, the green boxplots represent the “time to intubate” and the red boxplots represent the “time to ventilate”.

The recorded intubation times are shown graphically in Fig 3. Objective and subjective findings are summarized in Table 2.

**Table 2:** Intubation data of anesthetists with extensive previous experience in conventional and video laryngoscopy.

		Macintosh	Glidescope^®^ Go^™^	Dahlhausen VL	King Vision^™^	I-View^™^
Overall success rate, n/N (%)		56/58 (96.6) ^§§§^	56/58 (96.6) ^⸸⸸⸸^	58/58 (100) ^‡‡‡^	58/58 (100) ^⁑⁑⁑^	46/58 (79.3)
Time to vocal cords (s), median (IQR)		10.5 (6.8–16.1) ^§§§^	7 (5.3–10.3) ^###^ ^⸸⸸⸸^	6.5 (5.1–8.8) ^†††^ ^‡‡‡^	6.2 (4.2–9.5) ***^⁑⁑⁑^	19.8 (12–29.8)
Time to intubate (s), median (IQR)		20.9 (13.3–28) ^§§^	17.1 (13.9–26.9) ^⸸⸸⸸^	17.7 (13.1–23) ^‡‡^	18 (13.8–25.2) ^⁑⁑⁑^	28.4 (23.9–37.8)
Time to ventilate (s), median (IQR)		28.9 (20.4–42) ^§§^	27.2 (22–42.7) ^⸸⸸⸸^	27.8 (23.2–35.3) ^‡^	27.7 (23–38.5) ^⁑⁑⁑^	42.1 (32.7–50.1)
Number of intubation attempts, n (%)						
1		52 (89.7)	55 (94.8)	56 (96.6)	54 (93.1)	46 (79.3)
2		5 (8.6)	1 (1.7)	2 (3.4)	4 (6.9)	5 (8.6)
≥3		1 (1.7)	2 (3.4)	0 (0)	0 (0)	7 (12.1)
Median (IQR)		1 (1–1)	1 (1–1)	1 (1–1) ^‡^	1 (1–1)	1 (1–1)
Severity of dental compression, n (%)						
0		39 (67.2)	53 (91.4)	51 (87.9)	55 (94.8)	32 (55.2)
1		6 (10.3)	2 (3.4)	4 (6.9)	3 (5.2)	10 (17.2)
≥2		13 (22.4)	3 (5.2)	3 (5.2)	0 (0)	16 (27.6)
Median (IQR)		0 (0–1)	0 (0–0) ^##^ ^⸸⸸⸸^	0 (0–0) ^†^ ^‡‡^	0 (0–0) ***^⁑⁑⁑^	0 (0–2)
Number of optimization maneuvers, n (%)						
0		52 (89.7)	57 (98.3)	55 (94.8)	57 (98.3)	49 (84.5)
1		5 (8.6)	0 (0)	3 (5.2)	1 (1.7)	6 (10.3)
≥2		1 (1.7)	1 (1.7)	0 (0)	0 (0)	3 (5.2)
Median (IQR)		0 (0–0)	0 (0–0)	0 (0–0)	0 (0–0)	0 (0–0)
View (cm), median (IQR)		5.5 (2.4–8) ^§§^	1 (1–3) ^###^ ^⸸⸸⸸^ ^⁋^	2 (1–4) ^†††^ ^‡‡‡^	3 (1–4) *** ^⁑⁑⁑^	8 (6–9)
Handling (cm), median (IQR)		1 (0–3) ^§§§^	2 (1–3) ^⸸⸸⸸^	2 (1–4) ^‡‡‡^	2 (1–4) ^⁑⁑⁑^	7 (4.8–8)
Stability (cm), median (IQR)		4 (2–6.5)	2 (1–3) ^###^ ^⸸⸸⸸^	2.3 (1–4) ^††^ ^‡‡^	3 (2–4) * ^⁑^	4 (2.9–6)
Force applied during tracheal intubation (cm), median (IQR)		6 (4–8)	2 (2–3.3) ^###^ ^⸸⸸⸸^	3 (1–4) ^†††^ ^‡‡‡^	2.5 (2–4) *** ^⁑⁑⁑^	7 (5–9)
Difficulty of tracheal intubation (cm), median (IQR)		5.5 (3–7) ^§§§^	2 (1–3.6) ^###^ ^⸸⸸⸸^	2 (1–4.3) ^†††^ ^‡‡‡^	2.8 (2–4) ***^⁑⁑⁑^	7 (6–8)
Cormack-Lehane score, n (%)						
1		7 (12.1)	44 (75.9)	35 (60.3)	39 (67.2)	10 (17.2)
2		34 (58.6)	12 (20.7)	19 (32.8)	17 (29.3)	30 (51.7)
3		12 (20.7)	0 (0)	2 (3.4)	0 (0)	13 (22.4)
4		5 (8.6)	1 (1.7)	1 (1.7)	1 (1.7)	4 (6.9)
n.a.		0 (0)	1 (1.7)	1 (1.7)	1 (1.7)	1 (1.7)
Median (IQR)		2 (2–3)	1 (1–1) ^###^ ^⸸⸸⸸^	1 (1–2) ^†††^ ^‡‡‡^	1 (1–2) ***^⁑⁑⁑^	2 (2–3)
Preferred laryngoscope, n/N (%)	n.a. 2/58 (3.4)	5/58 (8.6)	23/58 (39.7)	18/58 (31)	10/58 (17.2)	0/58 (0)

Data are presented as median (inter-quartile range, IQR), number, n (%) or as fraction, n/N (%). Subjective findings are presented as numeric rating scale values (0 to 10 cm, from excellent/very easy to poor/very difficult).

^§§^ p < 0.01 Macintosh vs. Intersurgical

^§§§^ p < 0.001 Macintosh vs. Intersurgical

^##^ p < 0.01 Glidescope Go vs. Macintosh

^###^ p < 0.001 Glidescope Go vs. Macintosh

^⸸⸸⸸^ p < 0.001 Glidescope Go vs. Intersurgical

^⁋^ p < 0.05 Glidescope Go vs. King Vision

^†^ p < 0.05 Dahlhausen VL vs. Macintosh

^††^ p < 0.01 Dahlhausen VL vs. Macintosh

^†††^ p < 0.001 Dahlhausen VL vs. Macintosh

^‡^ p < 0.05 Dahlhausen VL vs. Intersurgical

^‡‡^ p < 0.01 Dahlhausen VL vs. Intersurgical

^‡‡‡^ p < 0.001 Dahlhausen VL vs. Intersurgical

* p < 0.05 King Vision vs. Macintosh

*** p < 0.001 King Vision vs. Macintosh

^⁑^ p < 0.05 King Vision vs. Intersurgical

^⁑⁑⁑^ p < 0.001 King Vision vs. Intersurgical.

### Paramedics with little previous experience in ETI

We could not find a significant difference in “the time to intubate” between the conventional standard Macintosh laryngoscope, the Dahlhausen VL, the King Vision^™^ and the I-View^™^. However, the Glidescope^®^ Go^™^ enabled a significantly shorter “time to intubate” compared to the King Vision^™^ (7.6 s [2.5-12.7 s], p < 0.05). The “time to vocal cords” was significantly increased using the I-View^™^ compared to the other VLs (9.7 s [6.3-13.2 s], p < 0.001, King Vision^™^ vs. I-View^™^; 7.9 s [3.8-11.9 s], p < 0.05, Glidescope^®^ Go^™^ vs. I-View^™^; 7.1 s [3.2-10.9 s], p < 0.05, Dahlhausen VL vs. I-View^™^). Although, post-hoc analysis revealed no differences in “the time to ventilate” and the number of intubation attempts, the failure rate was 35.2% with the I-View^™^, 14.8% with the Macintosh laryngoscope, 9.3% with the King Vision^™^ and only 3.7% with the Glidescope^®^ Go^™^ and the Dahlhausen VL. However, post-hoc analysis revealed statistical significance only for the I-View^™^ (n/N (%):35/54 (64.8), p < 0.001, Glidescope^®^ Go^™^, Dahlhausen VL and King Vision^™^ vs. I-View^™^, p < 0.01 Macintosh laryngoscope vs. I-View^™^). The severity of dental compression was lower with the Glidescope^®^ Go^™^ and the King Vision^™^ compared to the I-View^™^ (0.8 [0.4-1.1], p < 0.001, King Vision^™^ vs. I-View^™^; 0.6 [0.4-0.9], p < 0.01, Glidescope^®^ Go^™^ vs. I-View^™^). The King Vision^™^ also caused less dental trauma than the use of the Macintosh laryngoscope and the Dahlhausen VL (0.4 [0.2-0.7], p < 0.01, King Vision^™^ vs. Macintosh laryngoscope; 0.6 [0.3-0.8], p < 0.01, King Vision^™^ vs. Dahlhausen VL). In addition, the number of optimization maneuvers was lower when using the Glidescope^®^ Go^™^ compared to the I-View^™^ (0.2 [0.1-0.4], p < 0.05). The Glidescope^®^ Go^™^, the Dahlhausen VL and the King Vision^™^ significantly improved the Cormack and Lehane grade compared to the Macintosh laryngoscope and the I-View^™^, when used by paramedics with low experience in video laryngoscopy (1 [0.6-1.4], p < 0.001, Glidescope^®^ Go^™^ vs. I-View^™^; 0.6 [0.3-0.8], p < 0.01, Glidescope^®^ Go^™^ vs. Macintosh laryngoscope; 1 [0.7-1.4], p < 0.001, Dahlhausen VL vs. I-View^™^; 0.6 [0.4-0.9], p < 0.001, Dahlhausen VL vs. Macintosh laryngoscope; 1 [0.6-1.3], p < 0.001, King Vision^™^ vs. I-View^™^; 0.5 [0.3-0.8], p < 0.01, King Vision^™^ vs. Macintosh laryngoscope).

Regarding the subjective values, the Glidescope^®^ Go^™^, the Dahlhausen VL and the King Vision^™^ enabled a significant better view (2.7 cm [1.8-3.6 cm], p < 0.001, Glidescope^®^ Go^™^ vs. Macintosh laryngoscope; 5.4 cm [4.5-6.4 cm], p < 0.001, Glidescope^®^ Go^™^ vs. I-View^™^; 2.4 cm [1.6-3.2 cm], p < 0.001, Dahlhausen VL vs. Macintosh laryngoscope; 5.2 cm [4.4-6 cm], p < 0.001, Dahlhausen VL vs. I-View^™^; 2.6 cm [1.8-3.4 cm], p < 0.001, King Vision^™^ vs. Macintosh laryngoscope; 5.4 cm [4.5-6.3 cm], p < 0.001, King Vision^™^ vs. I-View^™^) and required less force during tracheal intubation (2.2 cm [1.4-3.1 cm], p < 0.001, Glidescope^®^ Go^™^ vs. Macintosh laryngoscope; 2.8 cm [1.9-3.7 cm], p < 0.001, Glidescope^®^ Go^™^ vs. I-View^™^; 2.2 cm [1.4-3.1 cm], p < 0.001, King Vision^™^ vs. Macintosh laryngoscope; 2.8 cm [2-3.6 cm], p < 0.001, King Vision^™^ vs. I-View^™^; 1.9 cm [1-2.9 cm], p < 0.01, Dahlhausen VL vs. I-View^™^; 1.4 cm [0.5-2.2 cm], p < 0.05, Dahlhausen VL vs. Macintosh laryngoscope) compared to the Macintosh laryngoscope and the I-View^™^. In addition, the ETI was judged to be easier using the Glidescope^®^ Go^™^, the Dahlhausen VL and the King Vision^™^ (2.1 cm [1.3-2.9 cm], p < 0.001, Glidescope^®^ Go^™^ vs. Macintosh laryngoscope; 4.3 cm [3.3-5.2 cm], p < 0.001, Glidescope^®^ Go^™^ vs. I-View^™^; 3.7 cm [2.8-4.6 cm], p < 0.001, Dahlhausen VL vs. I-View^™^; 1.5 cm [0.7-2.4 cm], p < 0.05, Dahlhausen VL vs. Macintosh laryngoscope; 3.6 cm [2.6-4.5 cm], p < 0.001, King Vision^™^ vs. I-View^™^; 1.4 cm [0.5-2.3 cm], p < 0.05, King Vision^™^ vs. Macintosh laryngoscope). The I-View^™^ VL was considered to be less stable (2 cm [1.1-2.8 cm], p < 0.001, Glidescope^®^ Go^™^ vs. I-View^™^; 1.9 cm [1-2.7 cm], p < 0.001, Dahlhausen VL vs. I-View^™^; 1.8 cm [1.1-2.5 cm], p < 0.001, King Vision^™^ vs. I-View^™^; 1.6 cm [0.7-2.5 cm], p < 0.01, Macintosh laryngoscope vs. I-View^™^) and more difficult to handle (p < 0.001) (3.7 cm [2.7-4.6 cm], p < 0.001, Glidescope^®^ Go^™^ vs. I-View^™^; 3.1 cm [2.2-4.1 cm], p < 0.001, Dahlhausen VL vs. I-View^™^; 2.9 cm [1.9-3.8 cm], p < 0.001, King Vision^™^ vs. I-View^™^; 2.3 cm [1.3-3.4 cm], p < 0.001, Macintosh laryngoscope vs. I-View^™^) when compared to the other intubation devices. Regarding the view (2.8 cm [1.7-3.8 cm], p < 0.001) and the difficulty of tracheal intubation (2.2 cm [1-3.3 cm], p < 0.01), the I-View^™^ was rated even worse than the Macintosh laryngoscope. The Glidescope^®^ Go^™^ was rated best and even easier to handle than the conventional Macintosh laryngoscope (1.3 cm [0.4-2.3 cm], p < 0.05).

Thirty-nine percent of the paramedics preferred the Glidescope^®^ Go^™^, followed by the King Vision^™^ (26%) and the Dahlhausen VL (22%). The standard Macintosh laryngoscope was preferred by 11% and only 2% of the paramedics with little previous experience in ETI would use the I-View^™^ VL.

**Fig 4:**
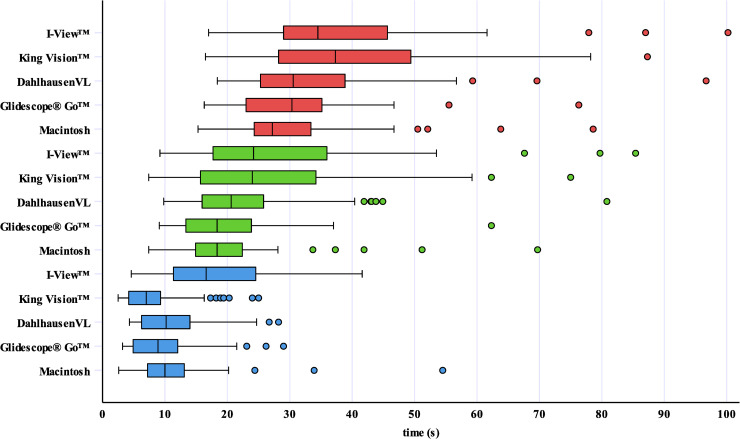
Recorded intubation times of the paramedics with little previous experience in conventional and even less previous experience in video laryngoscopy. Data are presented as boxplots that indicate the median (middle line), the 25^th^ and the 75^th^ percentile (box boundaries), the upper and lower limits within 1.5 times inter-quartile range (whiskers) and outliers (circles). The blue boxplots represent the “time to vocal cords”, the green boxplots represent the “time to intubate” and the red boxplots represent the “time to ventilate”.

The recorded intubation times are shown graphically in Fig 4. Objective and subjective findings are summarized in Table 3.

**Table 3:** Intubation data of paramedics with little previous experience in conventional and even less previous experience in video laryngoscopy.

		Macintosh	Glidescope^®^ Go^™^	Dahlhausen VL	King Vision^™^	I-View^™^
Overall success rate, n/N (%)		46/54 (85.2) ^§§^	52/54 (96.3) ^⸸⸸⸸^	52/54 (96.3) ^‡‡‡^	49/54 (90.7) ^⁑⁑⁑^	35/54 (64.8)
Time to vocal cords (s), median (IQR)		10 (7.2–13.1)	8.9 (4.8–12.1) ^⸸^	10.2 (6.2–14.1) ^‡^	7 (4.2–9.5) ^⁑⁑⁑^	16.6 (11.2–24.8)
Time to intubate (s), median (IQR)		18.4 (14.7–23.1)	18.4 (13.3–23.9) ^‖^	20.6 (15.9–26.2)	24 (15.3–35.5)	24.2 (17.3–36.3)
Time to ventilate (s), median (IQR)		27.2 (24–33.4)	30.4 (23–35.2)	30.6 (25.3–39.1)	37.3 (28–49.8)	34.5 (28.9–46)
Number of intubation attempts, n (%)						
1		47 (87)	53 (98.1)	52 (96.3)	46 (85.2)	43 (79.6)
2		3 (5.6)	0 (0)	0 (0)	4 (7.4)	9 (16.7)
≥3		4 (7.4)	1 (1.9)	2 (3.7)	4 (7.4)	2 (3.7)
Median (IQR)		1 (1–1)	1 (1–1)	1 (1–1)	1 (1–1)	1 (1–1)
Severity of dental compression, n (%)						
0		25 (46.3)	36 (66.7)	22 (40.7)	39 (72.2)	17 (31.5)
1		12 (22.2)	6 (11.1)	11 (20.4)	7 (13)	10 (18.5)
≥2		17 (31.5)	12 (22.2)	21 (38.9)	8 (14.8)	27 (50)
Median (IQR)		1 (0–2)	0 (0–1) ^⸸⸸^	1 (0–2)	0 (0–1) ** ^⁑⁑⁑^ ^⁋⁋^	1.5 (0–2)
Number of optimization maneuvers, n (%)						
0		46 (85.2)	53 (98.1)	48 (88.9)	51 (94.4)	42 (77.8)
1		5 (9.3)	1 (1.9)	6 (11.1)	0 (0)	10 (18.5)
≥2		3 (5.6)	0 (0)	0 (0)	3 (5.6)	2 (3.7)
Median (IQR)		0 (0–0)	0 (0–0) ^⸸^	0 (0–0)	0 (0–0)	0 (0–0)
View (cm), median (IQR)		4 (2.4–7) ^§§§^	1 (0.4–2) ^###^ ^⸸⸸⸸^	1.3 (1–2) ^†††^ ^‡‡‡^	1 (1–2) *** ^⁑⁑⁑^	8.9 (5–9.5)
Handling (cm), median (IQR)		3 (1.7–5) ^§§§^	2 (1–3) ^#^ ^⸸⸸⸸^	2.5 (1–4) ^‡‡‡^	2 (1–4) ^⁑⁑⁑^	7 (3–8.6)
Stability (cm), median (IQR)		2 (1–3) ^§§^	1 (0.9–3) ^⸸⸸⸸^	1 (1–3) ^‡‡‡^	2 (1–3) ^⁑⁑⁑^	3.5 (2–5)
Force applied during tracheal intubation (cm), median (IQR)		5 (3–7)	2 (1.4–3.5) ^###^^⸸⸸⸸^	3 (2–5) ^†^ ^‡‡^	2 (1.2–4) *** ^⁑⁑⁑^	5 (3–8)
Difficulty of tracheal intubation (cm), median (IQR)		4 (2.9–6) ^§§^	2 (1–3) ^###^^⸸⸸⸸^	2.5 (1–4) ^†^ ^‡‡‡^	2 (1–4.3) * ^⁑⁑⁑^	7 (3.9–9)
Cormack-Lehane score, n (%)						
1		26 (48.1)	46 (85.2)	46 (85.2)	45 (83.3)	25 (46.3)
2		19 (35.2)	7 (13)	8 (14.8)	6 (11.1)	9 (16.7)
3		5 (9.3)	0 (0)	0 (0)	3 (5.6)	5 (9.3)
4		4 (7.4)	1 (1.9)	0 (0)	0 (0)	15 (27.8)
Median (IQR)		2 (1–2)	1 (1–1) ^##^ ^⸸⸸⸸^	1 (1–1) ^†††^ ^‡‡‡^	1 (1–1) ** ^⁑⁑⁑^	2 (1–4)
Preferred laryngoscope, n/N (%)		6/54 (11.1)	21/54 (38.9)	12/54 (22.2)	14/54 (25.9)	1/54 (1.9)

Data are presented as median (inter-quartile range, IQR), number, n (%) or as fraction, n/N (%). Subjective findings are presented as numeric rating scale values (0 to 10 cm, from excellent/very easy to poor/very difficult).

^§§^ p < 0.01 Macintosh vs. Intersurgical

^§§§^ p < 0.001 Macintosh vs. Intersurgical

^#^ p < 0.05 Glidescope Go vs. Macintosh

^##^ p < 0.01 Glidescope Go vs. Macintosh

^###^ p < 0.001 Glidescope Go vs. Macintosh

^⸸^ p < 0.05 Glidescope Go vs. Intersurgical

^⸸⸸^ p < 0.01 Glidescope Go vs. Intersurgical

^⸸⸸⸸^ p < 0.001 Glidescope Go vs. Intersurgical

^‖^ p < 0.05 Glidescope Go vs. King Vision

^†^ p < 0.05 Dahlhausen VL vs. Macintosh

^†††^ p < 0.001 Dahlhausen VL vs. Macintosh

^‡^ p < 0.05 Dahlhausen VL vs. Intersurgical

^‡‡^ p < 0.01 Dahlhausen VL vs. Intersurgical

^‡‡‡^ p < 0.001 Dahlhausen VL vs. Intersurgical

* p < 0.05 King Vision vs. Macintosh

** p < 0.01 King Vision vs. Macintosh

*** p < 0.001 King Vision vs. Macintosh

^⁑⁑⁑^ p < 0.001 King Vision vs. Intersurgical

^⁋⁋^ p < 0.05 King Vision vs. Dahlhausen VL.

## Discussion

In the in-hospital setting, video laryngoscopy has been shown to reduce the number of failed intubations, to improve the glottic view and to reduce airway trauma [11]. However, there are only a few and heterogeneous studies comparing video laryngoscopy and direct laryngoscopy in the pre-hospital setting [19]. In addition, in the pre-hospital emergency setting, the intubation success depends not only on the laryngoscope type used, but also on provider training and its experience with the device. Bernhard and colleagues reviewed that paramedics tend to have lower success rates than physicians [20]. Thus, the aim of our prospective randomized crossover study was to compare the efficacy of different VLs with disposable blades and the conventional standard Macintosh laryngoscope, when used by anesthetists with extensive previous experienced in conventional and video laryngoscopy and paramedics with little previous experience in conventional laryngoscopy and even less previous experience in the use of video laryngoscopy in a simulated difficult airway scenario.

The results of our study suggest that in the hands of both anesthetists with extensive previous experience in ETI and paramedics with little experience in ETI, the Glidescope^®^ Go^™^, the Dahlhausen VL and the King Vision^™^ provide superior intubation conditions and can facilitate ETI in our simulated difficult airway scenario. In addition, the Glidescope^®^ Go^™^, the Dahlhausen VL and the King Vision^™^ improved the Cormack and Lehane grade when compared with the Macintosh laryngoscope. The design of these VLs offers a ‘view around the corner’ and provide an optimal glottis visualization via the camera, without the need to align the oral, pharyngeal and tracheal axes [21]. Despite the subjective advantages of the Glidescope^®^ Go^™^, the Dahlhausen VL and the King Vision^™^ we could not find a significant difference in the “time to intubate”, the “time to ventilate”, the number of intubation attempts and the number of optimization manoeuvers compared to the standard Macintosh laryngoscope with single-use blade when used by both anesthetists and paramedics. This is in line with the findings of Lewis and colleagues. In a Cochrane review including 64 studies with 7044 participants they found no evidence to indicate that the use of a VL would result in fewer intubation attempts. In addition, due to an extremely high level of statistical heterogeneity in terms of intubation times, Lewis and colleagues also found no evidence that using a VL would affect the time required for intubation [11].

In the hands of anesthetists with extensive previous experience in ETI, the Glidescope^®^ Go^™^, the Dahlhausen VL and the King Vision^™^ enabled a significantly shorter “time to vocal cords” and provided improved glottis exposure. However, we did not observe a statistically significant difference between the use of video laryngoscopy and direct laryngoscopy with regard to the overall success rate. This may be due to the anesthetists’ extensive previous experience in direct laryngoscopy with the conventional standard Macintosh laryngoscope in daily practice. Anesthetists experienced in direct laryngoscopy can identify landmarks of the anatomy and may be able to perform successful tracheal intubation even in case of worse Cormack and Lehane grade. Nonetheless, using the Macintosh laryngoscope, a greater peak force is required to align the axes and to visualize the glottis in a difficult airway [22, 23]. This is consistent with our subjective findings and may explain the lower rate of dental trauma when using the Glidescope^®^ Go^™^, the Dahlhausen VL and the King Vision^™^.

In the hands of paramedics with little previous experience in ETI, video laryngoscopy decreased the failure rate for tracheal intubation from 14.8% with the Macintosh laryngoscope to 3.7% with the Glidescope^®^ Go^™^ and the Dahlhausen VL. This corresponds to an absolute risk reduction of 11.1%. Although, this did not reach statistical significance, this result is in line with the findings of Savino and colleagues. In a systematic review and meta-analysis, including eight pre-hospital studies, they conclude that video laryngoscopy may lead to increased overall success rate in those emergency medical systems in which providers have less experience with ETI [19].

In our study, however, not all VLs performed equally. In the hands of both anesthetists with extensive previous experience and paramedics with little previous experience in ETI, the I-View^™^ VL was considered less stable and the most difficult to handle compared to the other intubation devices. The view was described as significantly worse and the intubation was rated more difficult. The worse performance may be due to the design of the I-View^™^ VL. In contrast to the Glidescope^®^ Go^™^, the Dahlhausen VL and the King Vision^™^ with hyperangulated disposable blades, the blade of the I-View^™^ one-size single-use VL has a standard Macintosh shape and is significantly longer. In a randomized crossover manikin study, Eismann and colleagues demonstrated, that the hyperangulated blade geometries of VLs provided a better view in difficult airway than the standard geometry of the Macintosh-type blade [24]. In addition, Schieren and colleagues showed that VLs with hyperangulated blades needed less force for intubation in difficult airways compared to Macintosh shaped laryngoscope blades [25]. The oversized length and the Macintosh shape of the blade could explain the significantly decreased overall success rate and higher rate of dental trauma when using the I-View^™^ VL.

In our prospective randomized crossover study, the Glidescope^®^ Go^™^ was rated best. It was considered to be even easier to handle than the Macintosh laryngoscope and enabled a significantly shorter “time to intubate” compared to the King Vision^™^ when used by paramedics with little previous experience in ETI. In the hands of experienced anesthetists, the Glidescope^®^ Go^™^ offered an even better view than the King Vision. The overall preference of the participants confirmed these results. Forty percent of the anesthetists and thirty-nine percent of the paramedics preferred the Glidescope^®^ Go.

## Limitations

The results of our study must be interpreted with consideration of several limitations. First, this study was performed using a high fidelity simulator and not on patients. Despite of their quality, manikins do not fully resemble human structures and are limited by their gross mechanics and tactile and textural differences. However, the use of anatomically correct manikins has proven to be a reliable surrogate for the clinical context [26]. In our difficult airway scenario, we observed a failure rate of 14.8% for paramedics using the Macintosh laryngoscope. This is in line with the findings of Lossius and colleagues. In their comprehensive meta-analysis they reported a failure rate of 15% in emergency services manned by non-physicians [4]. This indicates a good comparability and validity of our manikin. Second, some potential for bias exists, as it was not possible to blind the participants or the assessors to the device used in the difficult airway scenario. Third, the participants were aware that their performance was being assessed, which could lead to an altered performance due to the Hawthorne effect [27]. Fourth, certain measurements used in this study, such as grading the difficulty of tracheal intubation, have a subjective nature. Fifth, we examined only one difficult intubation scenario. The performance of the devices might be different in other scenarios (e.g. bloody/secretion-filled airway scenario, orofacial trauma scenario, obstructive oropharyngeal mass/tissue scenario). Sixth, a malleable intubation stylet was used to facilitate intubation with the Macintosh laryngoscope. Although, a malleable stylet allows to shape the endotracheal tube into an ideal position for direct laryngoscopy, the flexibility of the stylet might affect the performance of the Macintosh laryngoscope to some extent in the simulated difficult airway scenario. Seventh, although the hyperangulated VLs were associated with a statistically significant reduction in the “time to vocal cords” when used by experienced anesthetists, the clinical impact of this time difference remains uncertain. Eighth, the gender disparity in our study might have influenced the results. However, Waddington et al. could demonstrate that female and male intubators did not differ in their ability to intubate a Laerdal airway management trainer [28]. Finally, we compared only four different VLs with disposable blades and one standard Macintosh laryngoscope. There are other types of VLs with disposable blades and their utility in the management of the difficult airway in comparable settings might be different and should be investigated. In addition, Macintosh blades for direct laryngoscopy can vary substantially and therefore external validity may be limited to some extent.

## Conclusions

The use of video laryngoscopy did not affect the number of intubation attempts or the time required for successful ETI compared to direct laryngoscopy with a conventional standard Macintosh blade in a simulated difficult airway scenario. However, the Glidescope^®^ Go^™^, the Dahlhausen VL and the King Vision^™^ provided superior intubation conditions, enabled a better visualization of the glottis and thus facilitated ETI when used by both anesthetists with extensive previous experience in conventional and video laryngoscopy and paramedics with little previous experience in conventional and even less previous experience in video laryngoscopy. Although, the use of VLs did not affect the overall success rate of the anesthetists, video laryngoscopy in the hands of paramedics with little previous experience in ETI decreased the failure rate from 14.8% with the conventional standard Macintosh laryngoscope to 3.7% with the Glidescope^®^ Go^™^ and the Dahlhausen VL. In our manikin model of difficult airway, however, not all VLs performed equally well. The hyperangulated VLs performed significantly better than the Macintosh shaped I-View^™^, with the Glidescope^®^ Go^™^ being the most suitable VL.

Our results therefore suggest that hyperangulated VLs could be beneficial and might be the method of choice in comparable settings, especially for users with little previous experience in ETI. Further prospective randomized studies comparing direct laryngoscopy and video laryngoscopy in pre-hospital setting are necessary to confirm our findings.

## Supporting information

S1 Dataset (SAV)

S2 Dataset (SAV)
